# Design of Potent Inhibitors Targeting the Main Protease of SARS-CoV-2 Using QSAR Modeling, Molecular Docking, and Molecular Dynamics Simulations

**DOI:** 10.3390/ph16040608

**Published:** 2023-04-18

**Authors:** Mehdi Oubahmane, Ismail Hdoufane, Christelle Delaite, Adlane Sayede, Driss Cherqaoui, Achraf El Allali

**Affiliations:** 1 Laboratory of Molecular Chemistry, Department of Chemistry, Faculty of Sciences Semlalia, BP 2390, Marrakech 40000, Morocco; mehdi.oubahmane@ced.uca.ma (M.O.); ismail.hdoufane@edu.uca.ma (I.H.); cherqaoui@uca.ma (D.C.); 2 Laboratoire de Photochimie et d’Ingénierie Macromoléculaires (LPIM), Ecole Nationale Supérieure de Chimie de Mulhouse, Université de Haute-Alsace, 68100 Mulhouse, France; christelle.delaite@uha.fr; 3 University Artois, CNRS, Centrale Lille, University Lille, UMR 8181, Unité de Catalyse et Chimie du Solide (UCCS), F-62300 Lens, France; adlane.sayede@univ-artois.fr; 4 African Genome Center, Mohammed VI Polytechnic University, Ben Guerir 43150, Morocco

**Keywords:** SARS-CoV-2, main protease, QSAR, molecular docking, molecular dynamics simulations

## Abstract

Severe acute respiratory syndrome coronavirus 2 (SARS-CoV-2) infection is a serious global public health threat. The evolving strains of SARS-CoV-2 have reduced the effectiveness of vaccines. Therefore, antiviral drugs against SARS-CoV-2 are urgently needed. The main protease (Mpro) of SARS-CoV-2 is an extremely potent target due to its pivotal role in virus replication and low susceptibility to mutation. In the present study, a quantitative structure–activity relationship (QSAR) study was performed to design new molecules that might have higher inhibitory activity against SARS-CoV-2 Mpro. In this context, a set of 55 dihydrophenanthrene derivatives was used to build two 2D-QSAR models using the Monte Carlo optimization method and the Genetic Algorithm Multi-Linear Regression (GA-MLR) method. From the CORAL QSAR model outputs, the promoters responsible for the increase/decrease in inhibitory activity were extracted and interpreted. The promoters responsible for an increase in activity were added to the lead compound to design new molecules. The GA-MLR QSAR model was used to ensure the inhibitory activity of the designed molecules. For further validation, the designed molecules were subjected to molecular docking analysis and molecular dynamics simulations along with an absorption, distribution, metabolism, excretion, and toxicity (ADMET) analysis. The results of this study suggest that the newly designed molecules have the potential to be developed as effective drugs against SARS-CoV-2.

## 1. Introduction

Three years after the emergence of the COVID-19 disease caused by severe acute respiratory syndrome coronavirus 2 (SARS-CoV-2), the entire world is still suffering from the major health and socioeconomic consequences of the disease [1]. Accordingly, significant research efforts have been made to develop vaccines and drugs to effectively stop COVID-19. However, SARS-CoV-2 continues to claim human lives due to frequent mutations in its viral genome that make the new variants more transmissible and infectious, such as B.1.1.7 (alpha), B.1.351 (beta), B.1.617.2 (delta), and B.1.1.529 (omicron) [2,3,4,5]. Therefore, the discovery of new inhibitors that work better at different stages of SARS-CoV-2 propagation has become a priority.

SARS-CoV-2 is an enveloped positive-stranded RNA virus that, once it enters the cell, translates the single-stranded RNA into two large polyproteins known as pp1a and pp1ab that mediate viral replication and propagation [6]. During viral maturation, most cleavage events are controlled by a nonstructural protein 5 (nsp5), also known as the main protease (Mpro or 3CLpro), which is in charge of 11 different cleavages in the early stages of viral replication [7]. Mpro is an attractive target due to: (i) its pivotal role in viral replication and maturation [8]; (ii) minor mutations that have occurred in its genomic sequence in the new variants of SARS-CoV-2 compared to other major proteins, [9] and (iii) its inhibitors that are unlikely to be dangerous as no human proteases with similar cleavage specificity have been identified [10]. The Mpro monomer consists of three domains: domain I, domain II, and domain III, which include amino acid residues 8–101, 102–184, and 201–306, respectively [11]. Despite the fact that each individual monomeric component has its own active site, the Mpro of SARS-CoV-2 is enzymatically active only as a homodimer [11,12,13]. Therefore, it might be logical to block the catalytic site (CS) and dimer interface site (DIS) to inhibit the main function of the protease and its dimerization.

In continuation of our efforts to develop new potent Mpro inhibitors [14], in this study, two quantitative structure–activity relationships (QSAR) were developed based on the structural properties of dihydrophenanthrene derivatives and their inhibitory activities against Mpro. The first model, based on Monte Carlo optimization, was used to build SMILES-based QSAR models that provide insights into the design of new Mpro inhibitors. The second model used the genetic algorithm multi-linear regression approach (GA-MLR) to confirm the prediction of the inhibitory activity of the designed molecules. Since the dihydrophenanthrene derivative could inhibit SARS-CoV-2 by targeting the CS and DIS of the Mpro, a molecular docking study was carried out to investigate the interaction of the designed molecules within the CS and DIS of Mpro. In addition, the most potent designed molecules were subjected to molecular dynamics simulations in order to investigate their stability and behavior within the CS and DIS sites of Mpro. An absorption, distribution, metabolism, excretion, and toxicity (ADMET) evaluation of these molecules was also performed.

## 2. Results and Discussions

### 2.1. CORAL QSAR Model

**Figure 1:**
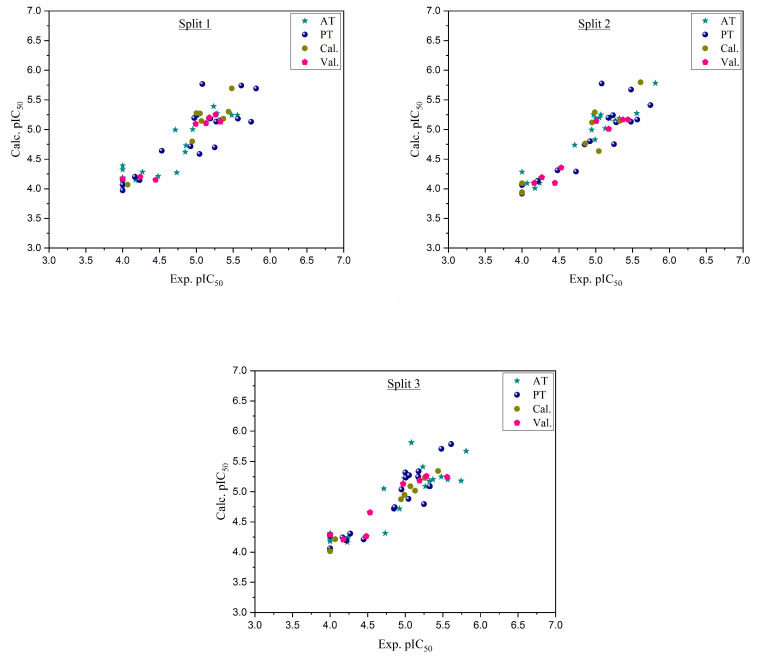
Graphical representation of experimental pIC_50_ versus calculated pIC_50_ for three splits.

Six QSAR models were built based on three random splits and two target functions: TF1 with W_IIC_ = 0 and TF2 with W_IIC_ = 0.2. Three splits were created from the entire dataset and each split was divided into four sets, as described below (for definitions of the parameters, refer to the Materials and Methods section (Section 3) of this article). The obtained statistical parameters for the developed SMILES-based QSAR models show that W_IIC_ = 0.2 increases the effect of IIC on Monte Carlo optimization. Table 1 displays the computed statistical parameters for all splits. Figure 1 depicts the experimental pIC_50_ values compared to the estimated values for the three splits. Table 1 clearly illustrates that all models are statistically reliable and fulfill the criteria set by Tropsha et al. [15] and Ojha et al. [16]. All data points of the three splits are in the applicability domain as shown in the Supplementary Materials in Table S1. The QSAR model with a higher R^2^ value for the validation set and a higher IIC value for the calibration set was selected as the leading model. Therefore, the QSAR model of split 2 had the highest R^2^ value of 0.9203 for the validation set and a higher IIC value of 0.9277 for the calibration set. The numerical values of the various parameters for the validation set of split 2 were Q^2^ = 0.8508, CCC = 0.9157, r^2^m = 0.8825, and Δr^2^m = 0.0647. The split 2 model is as follows in Equation (1):

(1) $${\mathrm{pIC}}_{50}=1.7915330\;\left(\pm 0.04504500\right)+0.0672532\;\left(\pm 0.0010114\right)\times \mathrm{DCW}\left(3,17\right)$$

### 2.2. GA-MLR QSAR Model

The GA-MLR method was applied to the training set and then evaluated to the test set using the six selected descriptors that contribute to the inhibitory activity (Table 2). The GA-MLR model Equation (2) and its statistical parameters are presented below:

(2) $$\begin{array}{cc} {\mathrm{pIC}}_{50}= & 0.5833\;\times \;\mathrm{Eig}09\text{\_}\mathrm{AEA}\left(\mathrm{dm}\right)\;-\;0.018\;\times \;P\text{\_}\mathrm{VSA}\text{\_}5\;-\;0.1949\;\times \;s3\text{\_}\mathrm{numSharedNeighbors}+\\ & 1.3785\;\times \;s3\text{\_}\mathrm{relPathLength}\text{\_}2+5.0014\;\times \;\mathrm{SpMin}2\text{\_}\mathrm{Bh}\left(m\right)\;-\;0.543\;\times \;\mathrm{GATS}5m; \end{array}$$

N_tr_ = 39; R^2^ = 0.9288; RMSE_tr_ = 0.1504; Q^2^_loo_ = 0.8902; R^2^_ext_ = 0.8558; Q^2^_F1_ = 0.8554; Q^2^_F2_ = 0.8554; Q^2^_F3_ = 0.8783; CCC_ext_ = 0.9228; s = 0.1660, where N_tr_ is the total samples in training and CCC represents the concordance correlation coefficient. Q^2^_F1_, Q^2^_F2_ and Q^2^_F3_ are external validation criteria.

**Figure 2:**
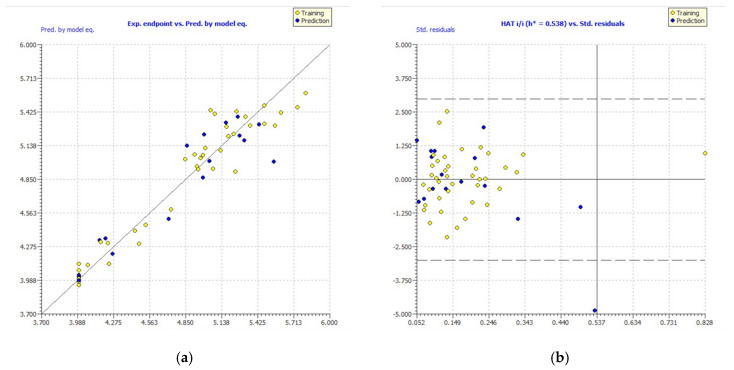
Experimental vs. predicted pIC_50_ values computed by GA-MLR (**a**). William’s plot (**b**).

The performance of the aforementioned parameters of the established GA-MLR model passes the OECD’s standard validation criteria. Furthermore, Figure 2a depicts the experimental pIC_50_ endpoints and the predicted endpoints by the constructed GA-MLR model, which demonstrates a good correlation between the studied activity and the six selected descriptors. The AD is used to analyze the space of the leading model in order to further validate the generated model. The AD was carried out using the leverage approach, as seen in the Williams plot in Figure 2b. The dashed lines represent the cutoff value of ±3 standard deviations, while the caution line for the X outlier (h*) is 0.538. All molecules in William’s plot fall within the AD, with the exception of one molecule.

### 2.3. Mechanistic Interpretation

A mechanistic interpretation is an essential component of the OECD. These models can be used to identify and evaluate the molecular properties that are responsible for increasing and decreasing an endpoint value. Multiple Monte Carlo optimization runs can be used to determine the mechanistic interpretation of the CORAL model. The chemical characteristics derived from the SMILES attributes with positive CWs are found to be promoters of an increase in the pIC_50_ value, whereas the SMILES attributes with negative CWs are shown to be promoters of a decrease in the pIC_50_ value in three independent Monte Carlo optimization runs. SMILES attributes containing both positive and negative CWs are undefined. Table 3 shows the primary promoters causing an increase or decrease in pIC_50_ values, along with the associated CWs, during three separate runs of the developed QSAR model for split 2.

**Figure 3:**
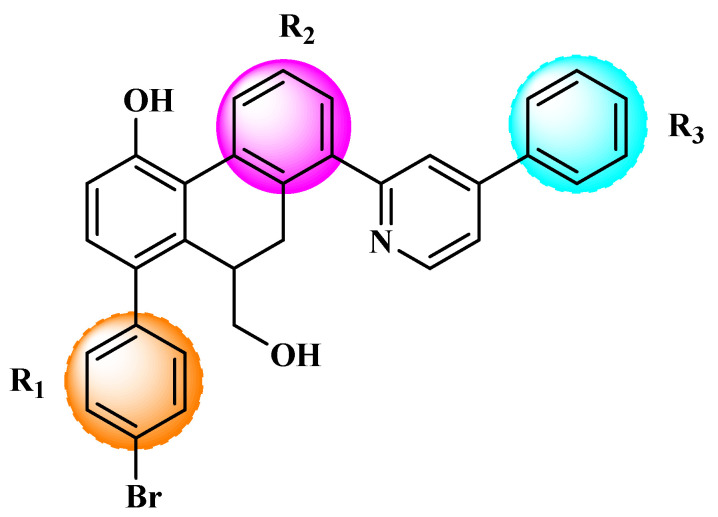
Chemical structures of the newly designed compounds.

Based on the findings listed in Table 3, the increase promoters were used and introduced into the lead compound (**49**), which has the highest pIC_50_ value, while the decrease promoters were avoided. The propagation promoters were examined at three distinct sites in the lead compound to design novel Mpro inhibitors (Figure 3). All the newly developed compounds, along with their chemical structures and the calculated pIC_50_ values by the two models, are listed in Table S2. The CORAL model predicted a pIC_50_ range of 5.82–6.44 for all the newly developed compounds. The GA-MLR QSAR model confirmed the inhibitory activity of the newly developed compounds, with the exception of **49v**, which exhibited a pIC_50_ value slightly lower than the one of the lead compound **49**.

### 2.4. Molecular Docking Analysis

To further validate the design strategy, a molecular docking approach was performed to investigate the ligand–protein interactions between the newly designed compounds with the reference lead compound (**49**) and the SARS-CoV-2 Mpro. The lead compound inhibits SARS-CoV-2 Mpro via mixed inhibition, which means that it could bind to the CS and DIS of the Mpro. All designed molecules were first docked to the catalytic site of Mpro. Then, the best three molecules bound to the CS were further docked to the DIS of Mpro.

#### 2.4.1. Molecular Docking within the CS of Mpro

**Figure 4:**
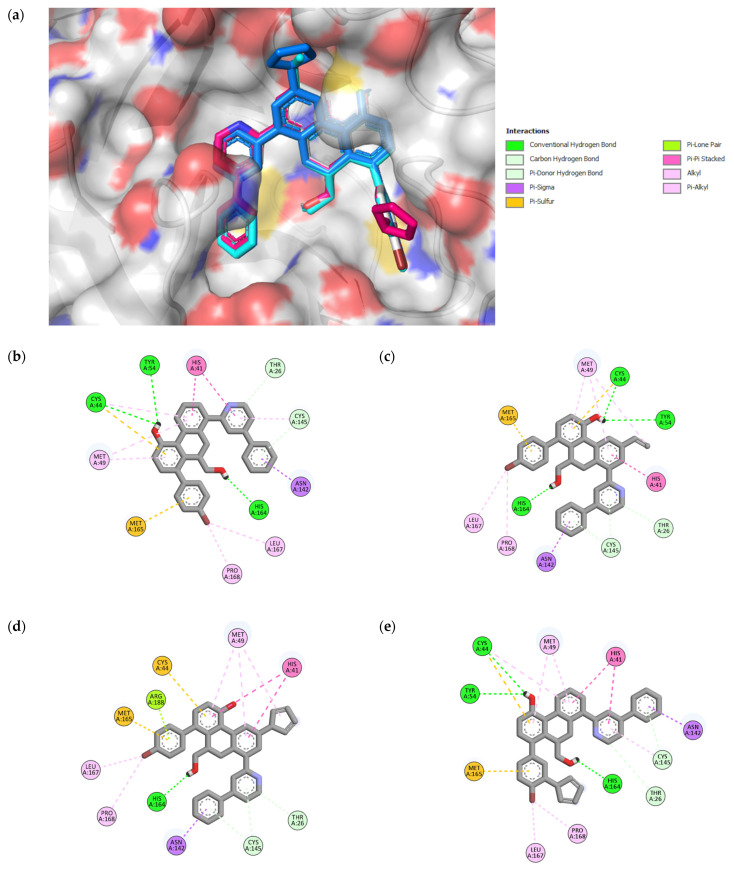
3D representation of superimposition of the binding poses within the CS of SARS-CoV-2 Mpro of **49**, **49n**, **49p**, and **49x** molecules in white, cyan, magenta and blue color respectively (**a**); 2D representation of interactions within the CS of SARS-CoV-2 Mpro of **49** (**b**), **49n** (**c**), **49p** (**d**), and **49x** (**e**) molecules.

To validate our docking protocol, a re-docking analysis of the co-crystallized ligand (3WL) with 6M2N was performed. The RMSD between the experimental and predicted poses of 3WL was determined to be 0.79 Å (Figure S1). This result shows that the docking protocol is appropriate for reproducing native poses (<2 Å). After validation of the parameters, a docking study was performed for all compounds against the Mpro protein. The binding affinity estimations are shown in Table S2. It was found that the binding affinity values of the designed molecules (except **49c** and **49k**) were higher compared to that of the compound **49** (−11.47 Kcal/mol) within the CS. The 2D representation of the best compounds (**49n**, **49p**, **49x**) bound with the CS of Mpro is shown in Figure 4. Their binding affinity values and interaction details are given in Table 4 and Table S3, respectively. The analysis of docking results shows that the lead compound **49** and the designed compound **49x** interacted with some important residues of the CS via five hydrogen bonds (one hydrogen bond with CYS145), nine hydrophobic (two of them with HIS41) and two pi–sulfur interactions. Compound **49n** was stabilized by the formation of six hydrogen bonds in which two hydrogen bonds were observed with CYS145, as well as two pi–sulfur interactions and seven hydrophobic interactions, where one of them interacted with HIS41. Compound **49p** interacted by four hydrogen bonds (two of them with CYS145), and eight hydrophobic (two of them formed with HIS41), along with two pi–sulfur and one pi–lone pair contacts. Analysis of the non-covalent interactions between the most potent designed molecules within the CS shows that they interact with important key binding residues, specifically the catalytic dyad (HIS41 and CYS145); thus, they can serve as important protease inhibitors.

#### 2.4.2. Molecular Docking within the DIS of Mpro

**Figure 5:**
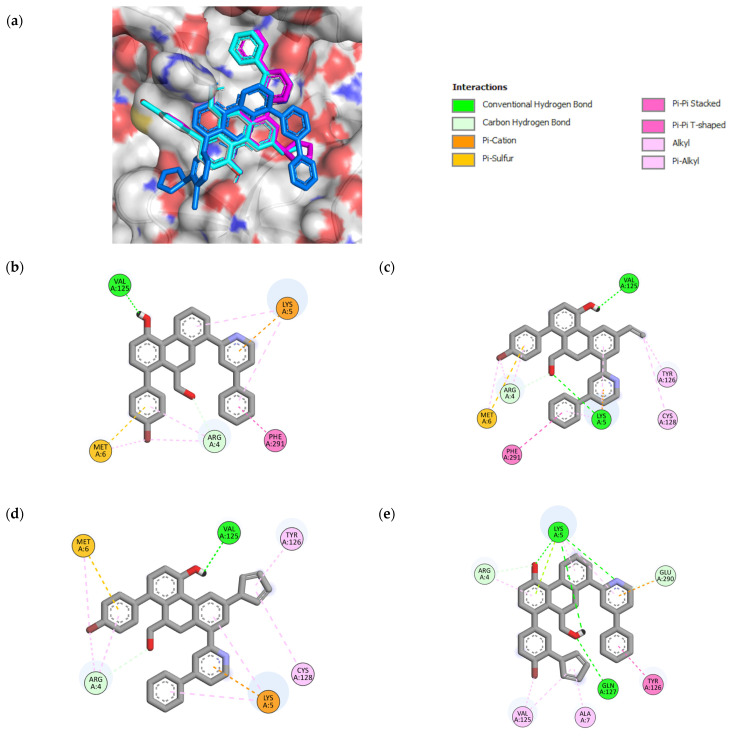
3D representation of superimposition of the binding poses within the DIS of SARS-CoV-2 of **49**, **49n**, **49p**, and **49x** molecules in white, cyan, magenta and blue color respectively (**a**); 2D representation of interactions within the DIS of SARS-CoV-2 Mpro of **49** (**b**), **49n** (**c**), **49p** (**d**), and **49x** (**e**) molecules.

After selecting the three best compounds for catalytic site inhibition, we performed a docking study of these compounds within the DIS of Mpro. The monomeric structure (6M2Q) was used as a receptor to avoid the structural changes that may occur in the dimeric structure of Mpro. The binding affinity and 2D representation of the ligand–protein interactions of the three compounds with the DIS of SARS-CoV-2 Mpro are shown in Table 4 and Figure 5, respectively. The interaction details are represented in Table S4. Compound 49 was stabilized within the DIS by two hydrogen bonds with Val125 and ARG4, one pi–sulfur interaction, one electrostatic interaction with LYS5, and seven hydrophobic interactions where five of them interacted with ARG4 and LYS5. The binding affinity values of −9.18, −9.95, and −9.06 Kcal/mol for compounds **49n**, **49p**, and **49x**, respectively, show that they bind better in the DIS than the lead compound **49** (−8.65 Kcal/mol). Compound **49n** was stabilized using three hydrogen bonds with VAL125, LYS5, and ARG4, one electrostatic interaction with LYS5, and one pi–sulfur interaction, along with five of nine hydrophobic interactions seen to be formed with ARG4 and LYS5. The interaction with compound **49p** was found to be stabilized by forming two hydrogen bonds with VAL125 and ARG4, one pi–sulfur interaction, one electrostatic interaction with LYS5, and eight hydrophobic interactions (five of them interacting with ARG4 and LYS5). Furthermore, compound **49x** was stabilized by six hydrogen binding interactions where five of them interacted with LYS5, ARG4, and GLU290, and four out of eight hydrophobic interactions were seen to be formed with ARG4 and LYS5, plus one pi–lone pair interaction with LYS5 and one electrostatic interaction with GLU290. Overall, the non-covalent interactions of the three compounds within the DIS of Mpro demonstrate that the compounds interacted with one of the important residues (ARG4, LYS5, and GLU290), which have been proposed to be involved in the dimerization of Mpro. This could be a potential strategy to disrupt the stability of the dimer interface and to prevent the formation of the dimeric structure of Mpro.

### 2.5. Molecular Dynamics Simulations (MDs) of Top-Three Designed Molecules

Molecular dynamics simulations (MDs) were performed to evaluate the conformational stability of ligand–protein complexes and the binding ability of ligands. In this study, the metrics RMSD, RMSF, and Rg were calculated. RMSD is a metric used to measure the deviation of a protein’s backbone from its initial conformation to its final conformation. The amount of deviation observed during a simulation is used to determine the stability of the protein with respect to its structural conformation. A protein that maintains a stable structure will have minimal deviation in its backbone, while a protein with a less stable structure will have greater deviation. RMSF analysis is used to identify the flexible regions of protein–ligand complexes. In proteins, regions such as loops, turns, and coils that are less organized have higher RMSF values, while more structured regions such as alpha helices and beta sheets have lower RMS fluctuations. The Rg is a measure of the compactness of the protein structure, which is determined by comparing the Rg values of the protein before and after the ligand binding.

#### 2.5.1. MDs of the Top-Three Designed Molecules within the CS of Mpro

**Figure 6:**
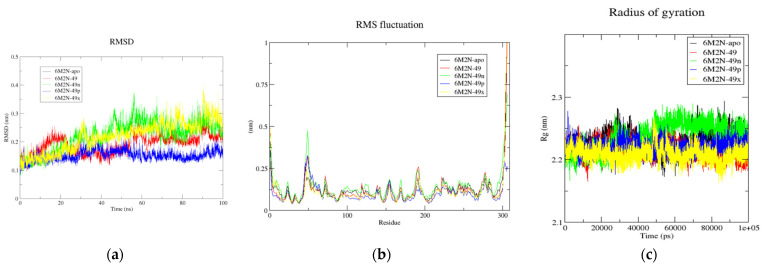
(**a**) Time-dependent RMSD of c-α backbone of the Mpro-apo, 6M2N-49, 6M2N-49n, 6M2N-49p, and 6M2N-49x. (**b**) The RMSF for c-α atoms of Mpro-apo, 6M2N-49, 6M2N-49n, 6M2N-49p, and 6M2N-49x. (**c**) Plot of Rg vs. time for Mpro-apo, 6M2N-49, 6M2N-49n, 6M2N-49p, and 6M2N-49x.

Analysis of the MDs results (Figure 6, Table 5) shows that the overall average RMSD values of 6M2N-apo, 6M2N-49, 6M2N-49n, 6M2N-49p, and 6M2N-49x are 0.220, 0.191, 0.221, 0.152, and 0.241 nm, respectively. The lowest average RMSD value indicates that 6M2N-49p exhibits greater stability compared to Mpro bound to compound **49**. On the other hand, the higher RMSD values indicate that 6M2N-49n and 6M2N-49x may have less stability compared to 6M2N-49p and 6M2N-49. Furthermore, the RMSF analysis of the complexes and the apo form of Mpro were computed against C-alpha atoms throughout the entire simulation. For the analysis of the three compounds binding to the Mpro, the RMSF values of the Mpro bound to compound **49** were used as a baseline for evaluating the flexibility of the designed compounds. The overall average RMSF value for 6M2N-apo, 6M2N-49, 6M2N-49n, 6M2N-49p, and 6M2N-49x was found to be 0.115, 0.113, 0.131, 0.092, and 0.103 nm, respectively. The 6M2N-49p complex confirms the RMSD results as it has the lowest RMSF compared to all systems. The 6M2N-49x complex showed a lower RMSF than the 6M2N-49 complex, suggesting that **49x**, like **49p**, could act as a potential inhibitor of Mpro at its catalytic site. In addition, the Rg plot for 6M2N-apo, 6M2N-49, 6M2N-49n, 6M2N-49p, and 6M2N-49x gave average Rg values of 2.23, 2.209, 2.231, 2.213, and 2.203 nm, respectively. The 6M2N-49x and 6M2N-49p complexes showed similar or slightly lower Rg values compared to the Mpro bound to compound **49**, indicating that these compounds are more tightly packed.

Finally, we performed the MM/GBSA analysis to calculate the interaction energies of the complexes for the entire MD trajectory. The results are shown in Table 6. The binding free energies for 6M2N-49n, 6M2N-49p, and 6M2N-49x are −19.63, −19.77, and −30.3 kcal mol-1, respectively, indicating higher binding affinity compared to the reference compound.

#### 2.5.2. MDs of the Top-Three Designed Molecules within the DIS of Mpro

**Figure 7:**
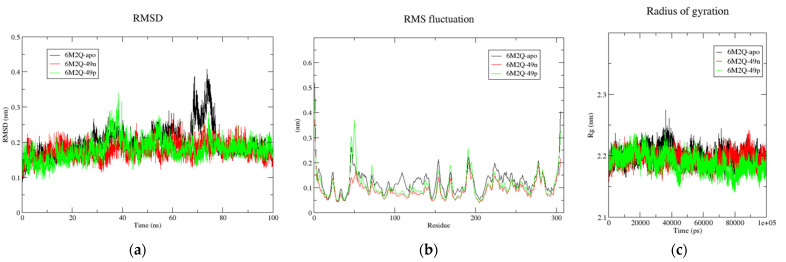
(**a**) Time-dependent RMSD of c-α backbone of the Mpro-apo, 6M2N-49n, and 6M2N-49p. (**b**) The RMSF for c-α atoms of Mpro-apo, 6M2N-49n, and 6M2N-49p. (**c**) Plot of Rg vs. time for Mpro-apo, 6M2N-49n, and 6M2N-49p.

MDs were performed to investigate the stability and conformational changes of the monomeric form of Mpro (6M2Q-apo) and the ligand–protein complexes 6M2Q-49, 6M2Q-49n, 6M2Q-49p, and 6M2Q-49x when the compounds were bound to the DIS of Mpro. After visualizing the dynamics of the compounds, we found that the reference compound **49** and **49x** left their binding site after 50 ns and 20 ns, respectively. In contrast, **49n** and **49p** remain bound throughout the simulation. Following these results, we present the RMSD, RMSF, and Rg plots for 6M2Q-apo, 6M2Q-49n, and 6M2Q-49p in Figure 7 and their overall average values in Table 7. The overall average RMSD values of 6M2Q-apo, 6M2Q-49n, and 6M2Q-49p were 0.198, 0.176, and 0.178 nm, respectively. Both 6M2Q-49n, and 6M2Q-49p showed lower RMSD values compared to 6M2Q-apo, suggesting greater stability of the ligand-Mpro complexes than Mpro-apo. In addition, RMSF analysis shows that the fluctuations were smaller than for the apo form of Mpro (RMSFavg = 0.116 nm) with average RMSF values of 0.087 nm and 0.102 nm for 6M2Q-49n and 6M2Q-49p, respectively. Furthermore, the Rg values for the ligand–protein complexes are lower than the apo form of Mpro, indicating that the ligands are more closely packed. These findings support the RMSD and RMSF results.

Considering all MD simulation results for the complexes with Mpro, **49n** and **49p** could lead to potentially higher activity because they have strong binding to both sites (CS and DIS) of Mpro, whereas **49x** is more stable when bounded to the CS.

### 2.6. Pharmacokinetic and Toxicity Predictions

The results of the ADMET analysis for the reference and three designed compounds were presented in Table 8. The human intestinal absorption (HIA) values reveal that these compounds have high absorption in the intestine, which makes them suitable for oral administration. With moderate predicted permeability through the blood-brain barrier (BBB), these compounds are expected to have acceptable bioavailability in the central nervous system. In addition, these compounds do not interfere with the normal functioning of cytochrome P450, which reduces the risk of unexpected or undesirable drug interactions. Moreover, the designed compounds do not exhibit toxic behavior, indicating that they may be suitable therapeutic candidates in pre-clinical trials.

## 3. Materials and Methods

### 3.1. Data Collection, Molecular Structures Preparation and Molecular Descriptor Calculation

For this study, the 2D structures of all 55 dihydrophenanthrene derivatives, collected from the published work of Jian-Wei Zhang & al [17], were sketched using ACD/ChemSketch software and converted to 3D structures using ChemDraw 16.0 software. For geometry optimization and partial charge assignment, the MMFF94 force field was employed with the steepest descent as an algorithm and with 1000 as the number of steps used for optimization. Then, the geometries were optimized using the AM1 method in the gas phase implemented in the Gaussian 09 software [18]. Frequency analysis was checked to investigate the energy minima of the optimized derivatives. The optimized molecular structures were converted into Simplified Molecular Input Line Entry System (SMILES) codes for the modeling process of the CORAL model. To build the GA-MLR model, alvaDesc software (version 1.0.8) was used to calculate the molecular descriptors using the optimized 3D structures [19]. To avoid multicollinear variables in the QSAR model, the number of generated variables was reduced by eliminating descriptors that had a constant value of over 95%, and by keeping only one of the descriptor pairs that possessed a correlation coefficient higher than 0.9. The experimental data value of each molecule (half-maximal inhibitory concentration, IC_50_) was converted into the form of a logarithm (−log IC_50_). The molecular structures and their corresponding IC_50_ data are listed in Table S5, and their SMILES notation and converted pIC_50_ are available in Table S6.

### 3.2. SMILES-Based QSAR Model Building

For QSAR modeling using CORAL 2019 software [20], the entire dataset (55 derivatives) was randomly divided into three splits. Each split consisted of 4 sets: active training (AT, 35%), passive training (PT, 35%), calibration (Cal, 15%), and validation (Val, 15%). Each set has a specific role in the development of the QSAR model, which is well explained in the literature [21,22,23]. The AT set is used to develop the model and generate correlation weights. These weights are then used to compute the descriptors for all the compounds involved in the modeling process. The PT set is used to evaluate the robustness of the model for compounds not belonging to those used to construct the model. The Cal set is used to detect the onset of overfitting. The Val set provides an independent evaluation of the statistical quality of the model using data for compounds that were not included in the model development and optimization. The distribution and identity level of compounds in three splits are shown in Table 9.

The development of QSAR models based on SMILES notation uses the optimal descriptors Equation (3).

(3) DSMILESCWT,Nepoch=∑CWSk+∑CWSSk+∑CWSSSk+ CWPAIR+CWHARD+CWCmax+CWOmax+CWNmax

^SMILES^DCW (T, N_epoch_) combines SMILES-based attributes associated with a correlation weight (CW). T and N_epoch_ are the thresholds and the number of epochs calculated by the Monte Carlo optimization method in the course of model building [24]. The comprehensive explanation of the SMILES attributes is provided in Table 10.

Following the calculation of all CWs, the linear regression technique was utilized to establish QSAR models, as indicated in Equation (4).

(4) pIC50=C0+C1×DSMILESCWT, Nepoch 

where *C*_0_ is the intercept, while *C*_1_ is the slope of the regression equation.

The target functions TF1 and TF2 were used to optimize the Monte Carlo method for QSAR modeling. The equilibrium of the correlation method was used to calculate TF1 (Equation (5)), while the index of ideality of correlation (IIC) [25] was added to TF1 to obtain the modified target function TF2 (Equation (6)).

(5) $$\mathrm{TF}1=R_{AT}+R_{PT}-\left|R_{AT}-R_{PT}\right|*0.1$$

(6) $$\mathrm{TF}2=\mathrm{TF}1+\mathrm{IIC}\;*\;W_{IIC}$$

(7) IIC=Rset×minM−AEcal,M+AEcalmaxM−AEcal,M+AEcal

R*_AT_*, R*_PT_*, and R*_set_* are the correlation coefficients between the observed pIC_50_ and the predicted pIC_50_ for the active training set, the passive training set, and for a given set, respectively. The mean absolute error (MAE) is determined as follows:

(8) M+AEcal=1N+∑k=1N+ΔkΔk≥0 ; N is the number of Δk≥0

(9) M−AEcal=1N−∑k=1N−ΔkΔk<0 ;N is the number of Δk<0 

To build robust QSAR models, the optimal threshold (T*) and number of epochs (N*) were calculated by analyzing the best statistical parameters for the calibration set. In the search for the best T* and N*, the ranges from 1 to 10 for the threshold and from 1 to 30 for the N_epoch_ were used for the optimization. Three optimization probes were used. The weight of IIC (W_IIC_) is an empirical coefficient; in this study, the W_IIC_ = 0 for TF1 and W_IIC_ = 0.2 for TF2.

### 3.3. GA-MLR QSAR Model Building

To initiate QSAR analysis, the initial stage is to select the most appropriate descriptors from the complete set of computed ones. For this purpose, alvaDesc software was used to compute 5666 molecular descriptors, and only 643 descriptors were filtered out based on the criteria mentioned above (Section 2.1). Then, a stepwise MLR method was performed using SPSS software to select the most relevant descriptors [26]. Finally, 6 molecular descriptors were kept. Using these selected descriptors, the MLR method established a linear relationship between the pIC_50_ endpoints of the molecules and their molecular descriptors by applying the ordinary least squares (OLS) algorithm implemented in the QSARINS software [27,28]. The data set was randomly divided into a training set (70%, 39 molecules) and a test set (30%, 16 molecules). The GA-MLR models were created with standard parameters except for subsets from 1 to 5, maximum generation of 10,000, and mutation probability of 0.05.

### 3.4. QSAR Models Validation

The validation step in QSAR is critical for evaluating the accuracy of the model in predicting the activity of new compounds. This step is critical for determining the robustness, reliability, and predictability of the QSAR model. There are four steps to validate the constructed model, including internal validation or cross-validation using the training data set, Y-randomization, external validation using the test data set, and evaluation of the applicability domain (AD) [29]. The validation steps and criteria of the GA-MLR and CORAL QSAR models are well explained in our previous articles [14,23,30,31].

### 3.5. Applicability Domain

The applicability domain (AD) resulting from the training data of a given in silico model was proposed by the Organization for Economic Co-operation and Development (OECD) guidelines [32]. AD can be used to assess the predictability of a created QSAR model for molecules that were not considered in the development of the model. AD is important to determine whether the created QSAR model interpolates (makes correct predictions) or extrapolates (makes less reliable predictions). Outliers are molecules that exist outside of the AD.

In the case of the SMILES-based QSAR, the statistical defects of SMILES are the guiding criteria for the definition of AD. The statistical defect (D) for a given molecule is the sum of the statistical defects, d(A), of all the attributes accessible in the SMILES notation, Equation (10).

(10) $$D=\mathrm{defect}\left(\mathrm{SMILES}\right)=\sum_{k=1}^{NA}d\left(A\right)=\sum_{k=1}^{NA}d(\frac{\left|P\left(A\right)-P^{\prime }\left(A\right)\right|}{N\left(A\right)-N^{\prime }\left(A\right)}$$

Here, P(A) and P′(A) represent the probabilities of the attributes (A) in the training set and calibration set, correspondingly, while N(A) and N’(A) indicate the frequencies of the attribute (A) occurring in the training set and calibration set, respectively.

A molecule is classified as an outlier if D > 2* $\bar{D}$, while a molecule is in the AD if D < 2*$\bar{D}$, where $\bar{D}$ is the estimated average for the AT, PT, and Cal sets [33].

The William plot of standardized residuals versus leverage was used to represent the AD in the GA-MLR model. Reliable model predictions have leverage values that are within ±3 standard deviations of the critical leverage and less than the warning leverage value h*. The molecules that fall outside the horizontal reference lines of the graph are outliers, while the influential molecules are those with h > h* [34].

### 3.6. Molecular Docking Study

In the current study, molecular docking was used to investigate the conformational patterns and ligand–protein interactions of the designed molecules within the CS and DIS sites of the SARS-CoV-2 Mpro. Two 3D crystallographic structures of the Mpro protein were downloaded from the Protein Data Bank (PDB) of the Research Collaboratory for Structural Biology (RCSB; https://www.rcsb.org/, accessed on 10 March 2023) with the PDB ID codes: 6M2N and 6M2Q [35]. The dimeric structure (6M2N) was used as a receptor molecule to explore the preferred conformation pose of the designed molecules in its active binding site, while the monomeric structure (6M2Q) was used to investigate the best conformational pose of the designed molecules in its DIS. Prior to the docking process, the two protein structures were prepared by removing water and heteroatom molecules, adding polar hydrogens and Gasteiger charges, and saving them in the pdbqt format with the assistance of AutodockTools. Using the same program, the 3WL, the lead compound **49**, and the designed molecules were produced and stored in the pdbqt format. The important residues within the CS and DIS sites were defined according to the literature in Table 11 [36,37]. Molecular docking simulations were performed using AutoDock 4.2 with the following parameters: (i) xyz coordinates of −33.162, −65.074, 41.434 and grid box dimensions of 40 × 0 × 40 for the CS, and (ii) xyz coordinates of 112.000, −17.078, 46.750 and grid box dimensions of 80×80×100 for the DIS. A grid spacing of 0.375 was set for both of the grid boxes. The Lamarckian Genetic Algorithm was used to generate 100 protein–ligand binding conformations for each molecule, with a maximum of 2,500,000 energy evaluations. The most reasonable binding configurations were then subjected to an evaluation that considered both ligand–receptor interactions and binding affinity. Discovery studio visualizer [38] and Pymol software [39] were used to analyze the docking outputs.

### 3.7. Molecular Dynamics Simulation Details

The newly designed compounds which showed stronger binding to the CS and DIS were subjected to all-atom molecular dynamics using the GROMACS 2020.6 (Groningen Machine for Chemical Simulation) software [40,41]. Before running the MD simulation, the CHARMM-GUI web server [42] was used to generate the initial input parameters implementing the CHARMM36 force field [43]. Based on a rectangular grid box, each complex was solvated within TIP3P water and the necessary counterions (Na^+^,Cl^-^) were added to maintain a salt concentration of 0.15 M through the Monte Carlo ion displacement. Energy minimization of each system was performed using the steepest descent algorithm with a maximum of 50,000 steps and a maximum force of 10.0 KJ/mol. The temperature and atmospheric pressure were set to 300 K and 1.01325 bar, respectively. Each system was equilibrated using canonical (NVT) and isothermal-isobaric (NPT) ensembles. After that, the MD simulation was performed for 100 ns. Based on the dynamics trajectory results, the root mean square deviation (RMSD), the radius of gyration (Rg), and root mean square flexibility (RMSF) were used to evaluate the structural stability of the designed molecules within the CS and DIS of Mpro. The obtained data were plotted using Xmgrace software [44].

### 3.8. MM-GBSA

The calculation of the binding free energies of protein–ligand complexes was estimated by the Molecular Mechanics Generalized Born Surface Area (MMGBSA) method using the gmx-mmgbsa package [45]. The binding free energy is calculated according to the following equations:

(11) $${\Delta G}_{bind}={\Delta G}_{complex}-\left[{\Delta G}_{protein}+{\Delta G}_{ligand}\right]\;\;\;\;\;\;\;\;\;\;\;\;\;\;\;\;\;\;\;\;\;\;$$

where the total free energy of the complex is represented by ΔG*_complex_*, while the energies of the isolated protein and ligand are represented by ΔG*_protein_* and ΔG*_ligand_*, respectively;

(12) $${\Delta G}_{bind}={\;\Delta E}_{gas}+{\Delta G}_{sol}={\Delta E}_{vdw}+{\Delta E}_{ele}+{\Delta G}_{polar}+{\Delta G}_{nonpolar}$$

where ∆E*_gas_* is the average potential energy of molecular mechanics in a vacuum, which includes both van der Waals interactions (∆E*_vdw_*) and electrostatic interactions (∆E*_ele_*). ∆G*_solv_* is the contribution to the solvation-free energy that is made up of the polar solvation energy (∆G*_polar_*) and the nonpolar solvation energy (∆G*_nonpolar_*).

### 3.9. ADMET Study

The absorption, distribution, metabolism, excretion, and toxicity (ADMET) analysis plays a critical role in drug discovery and development. Based on the ADMET results, we could predict the efficacy and safety of the potential hit compound in the early stages of drug development. In this regard, the ADMET predictions for the compounds were performed using the pkCSM server [46] for pharmacokinetic properties and the Osiris property explorer software [47] for toxicological properties.

## 4. Conclusions

The aim of this study was to find new inhibitors of SARS-CoV-2 replication by targeting the Mpro, a crucial protein involved in replicase polyprotein processing. To achieve this goal, we developed two QSAR models based on the structural properties of a series of dihydrophenanthrene derivatives that have shown potential inhibitory activity against Mpro. SMILES-based and GA-MLR QSAR models were built to provide insights in the design of novel potent Mpro inhibitors. Molecular docking and molecular dynamics simulations were further used as computational validation of the designed compounds. Overall, it has been shown that three compounds (**49n**, **49p**, and **49x**) exhibited a good inhibitory activity against Mpro. Also, they showed an important conformational and structural stability and a favorable binding profile. In addition, these drug candidates were found to be non-toxic and had acceptable pharmacological properties. By targeting the catalytic and dimer interface sites of Mpro, this suggests that these compounds have the potential to be developed as effective drugs against SARS-CoV-2. The combination of computational methods used in this study enabled the identification of new drug candidates, which can now be further evaluated for their efficacy and safety. Future studies could explore the feasibility of in vitro experiments to confirm and strengthen our computational modeling results. Nonetheless, the computational methods used in this study demonstrate their potential utility in accelerating drug discovery and directing future research directions against SARS-CoV-2.

## Figures and Tables

**Table 1:** Statistical parameters of built QSAR models and their corresponding equations.

Split	TF	Set	n	R^2^	CCC	IIC	Q^2^	Q^2^_F1_	Q^2^_F2_	Q^2^_F3_	Rm^2^_av_	ΔRm^2^	Crp^2^	Equation
**1**	**TF1**	AT	19	0.8031	0.8908	0.6518	0.7605						0.7855	**pIC_50_ = 1.8172593 (+− 0.0736101) + 0.0553060 (+− 0.0014479) × DCW(1,1)**
		PT	20	0.7237	0.8152	0.4176	0.6364						0.6964	
		Cal	8	**0.8085**	**0.8965**	**0.7468**	**0.5238**	0.8468	0.7854	0.8854	**0.7270**	**0.0438**	0.7259	
		Val	8	**0.7579**	**0.8490**	**0.6372**	**0.5803**				**0.6545**	**0.1869**		
	**TF2**	AT	19	0.8145	0.8978	0.8123	0.7734						0.7868	**pIC_50_ = 2.1090187 (+− 0.0660490) + 0.0635121 (+− 0.0016442) × DCW(5,14)**
		PT	20	0.7510	0.8619	0.6401	0.6889						0.7309	
		Cal	8	**0.8536**	**0.9182**	**0.9239**	**0.6657**	0.8743	0.8239	0.9060	**0.7874**	**0.1008**	0.7687	
		Val	8	**0.9161**	**0.9549**	**0.8433**	**0.8274**				**0.8771**	**0.0040**		
**2**	**TF1**	AT	20	0.9314	0.9645	0.6434	0.9187						0.8879	**pIC_50_ = 2.0227798 (+− 0.0329913) + 0.0786046 (+− 0.0009506) × DCW(7,5)**
		PT	19	0.8821	0.9245	0.4217	0.8428						0.8595	
		Cal	8	**0.8039**	**0.8857**	**0.6191**	**0.6788**	0.7331	0.7329	0.7594	**0.6830**	**0.1830**	0.6787	
		Val	8	**0.8506**	**0.8826**	**0.4703**	**0.7229**				**0.7827**	**0.1187**		
	**TF2**	AT	20	0.9191	0.9579	0.7844	0.9023						0.9029	**pIC_50_ = 1.7915330 (+− 0.0454500) + 0.0672532 (+− 0.0010114) × DCW(3,17)**
		PT	19	0.8283	0.9001	0.7511	0.7652						0.8001	
		Cal	8	**0.8612**	**0.9251**	**0.9277**	**0.7845**	0.8374	0.8373	0.8535	**0.7932**	**0.1285**	0.8266	
		Val	8	**0.9203**	**0.9157**	**0.6768**	**0.8508**				**0.8825**	**0.0647**		
**3**	**TF1**	AT	19	0.7424	0.8521	0.7754	0.6545						0.7102	**pIC50 = 1.9879653 (+− 0.1287947) + 0.0650533 (+− 0.0030713) × DCW(3,1)**
		PT	20	0.8129	0.8983	0.5442	0.7805						0.8008	
		Cal	9	**0.9736**	**0.9712**	**0.4888**	**0.9615**	0.9492	0.9483	0.9622	**0.8647**	**0.0379**	0.9167	
		Val	7	**0.8589**	**0.9068**	**0.7634**	**0.7030**				**0.6779**	**0.1627**		
	**TF2**	AT	19	0.7473	0.8554	0.7780	0.6645						0.6957	**pIC50 = 2.0784826 (+− 0.1186544) + 0.0690012 (+− 0.0031186) × DCW(10,7)**
		PT	20	0.8210	0.9039	0.5496	0.7899						0.7929	
		Cal	9	**0.9860**	**0.9864**	**0.9925**	**0.9700**	0.9758	0.9754	0.9820	**0.8849**	**0.0241**	0.8731	
		Val	7	**0.8728**	**0.9197**	**0.7740**	**0.7444**				**0.7169**	**0.1417**		

**Table 2:** Molecular descriptors and their description.

Molecular Descriptor	Description
Eig09_AEA(dm)	Eigenvalue n. 9 from augmented edge adjacency matrix weighted by dipole moment
P_VSA_MR_5	P_VSA-like on Molar Refractivity, bin 5
s3_numSharedNeighbors	Number of shared neighbours in substituent 3 with other substituents
s3_relPathLength_2	Maximum path length of the substituent 3 normed by s3_size
SpMin2_Bh(m)	Smallest eigenvalue n. 2 of Burden matrix weighted by mass
GATS5m	Geary autocorrelation of lag 5 weighted by mass

**Table 3:** Promoters of increase and decrease of pIC_50_ endpoint value from split 2 and their description.

	CWs Probe 1	CWs Probe 2	CWs Probe 3	N_AT_^a^	N_PT_^b^	N_Cal_^c^	Defect [SAk] ^d^	Comment
**Promoters of increase**								
(...........	0.21284	0.24591	0.24281	20	19	8	0.0000	Branching
(...O...(...	1.23889	1.32719	1.06767	20	19	8	0.0000	Two-sided branching of oxygen
++++N---O===	0.92838	1.33202	1.79720	20	19	8	0.0000	Presence of nitrogen with oxygen
1...........	0.30243	0.03065	0.22419	20	19	8	0.0000	Presence of one ring
C...........	0.01768	0.10610	0.24270	20	19	8	0.0000	Presence of sp3 carbon
c...(.......	0.10123	0.12192	0.30233	20	19	8	0.0000	Sp2 carbon with branching
c...(...c...	0.14109	0.27861	0.30707	20	19	8	0.0000	Branching between two sp2 carbons
c...........	0.57756	0.61985	0.68283	20	19	8	0.0000	Presence of a sp2 carbon
c...2.......	0.03199	0.01794	0.13095	20	19	8	0.0000	Presence of at least two aromatic rings/Presence of sp2 carbon with two rings
c...2...c...	0.64083	0.33025	0.27204	20	19	8	0.0000	Aromatic ring surrounded by two sp2 carbons
c...c...(...	0.45761	0.46821	0.64020	20	19	8	0.0000	Presence of two sp2 carbons with branching
c...c.......	0.09429	0.44519	0.11101	20	19	8	0.0000	Presence of two sp2 carbons
c...c...c...	0.28956	0.77633	0.66046	20	19	8	0.0000	Presence of three sp2carbons
C...C...(...	0.40471	0.84999	1.11520	19	19	8	0.0019	Presence of two sp3 carbons with branching
**Promoters of decrease**								
O...........	−0.96058	−1.30101	−1.49847	20	19	8	0.0000	Presence of oxygen
n........…	−1.27893	−1.65511	−1.62009	20	19	8	0.0000	Presence of sp2 nitrogen
n...c.......	−0.11779	−0.15247	−0.22729	20	19	7	0.0046	Presence of sp2 nitrogen with sp2 carbon
=……….	−0.52134	−0.04031	−0.07066	15	19	7	0.0057	Presence of double covalent bond
c...C.......	−0.28402	−0.70453	−0.44302	9	12	4	0.0038	Combination of sp2 carbon and sp3 carbon
c...(...C...	−0.24438	−0.23144	−0.63654	8	12	4	0.0083	Branching between sp2 carbon and sp3 carbon
C...O...C...	−0.19900	−0.49944	−0.58811	7	7	3	0.0025	Sp3 oxygen surrounded by two sp3 carbons

N_AT_ ^a^, N_PT_ ^b^, and N_Cal_ ^c^ represent the number of SMILES (samples) in AT, PT, and Cal sets, respectively, that comprise a particular attribute (SAk). Defect [SAk] ^d^ is calculated as the difference between the probability of SAk in the training and calibration sets divided by the total number of SAk in the training and calibration sets.

**Table 4:** The newly designed compounds and their predicted pIC_50_ using the Monte Carlo optimization and the GA-MLR models.

		Binding Affinity (Kcal/mol)	
Compounds	Chemical Structure	Catalytic Site	Dimer Interface Site
**49** (ref)	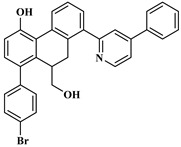	−11.47	−8.65
**49n**	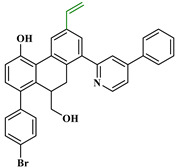	−12.63	−9.18
**49p**	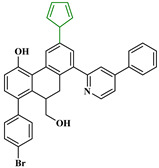	−12.98	−9.95
**49x**	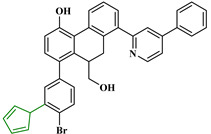	−12.85	−9.06

**Table 5:** The calculated average parameters for all the systems throughout 100 ns MD simulation run.

Systems	6M2N-Apo	6M2N-49	6M2N-49n	6M2N-49p	6M2N-49x
RMSD (nm)	0.220	0.191	0.221	0.152	0.241
RMSF (nm)	0.115	0.113	0.131	0.092	0.103
Rg (nm)	2.223	2.209	2.231	2.213	2.203

**Table 6:** Detailed binding free energy calculated by MM/GBSA for all complexes. All the values are given in kcal/mol.

Systems	6M2N-49	6M2N-49n	6M2N-49p	6M2N-49x
ΔEvdw	−31.98	−33.54	−40.95	−51.68
ΔEele	−11.93	−8.38	−16.3	−18.1
ΔEGB	31.1	26.48	42.76	45.97
ΔEsurf	−4.26	−4.19	−5.28	−6.5
ΔGgas	−43.91	−41.92	−57.25	−69.78
ΔGsolv	26.84	22.29	37.48	39.48
Δtotal	−17.07	−19.63	−19.77	−30.3

**Table 7:** The calculated average parameters for all the systems after 100 ns MD simulation run.

Systems	6M2Q-Apo	6M2Q-49n	6M2Q-49p
RMSD (nm)	0.198	0.176	0.178
RMSF (nm)	0.116	0.087	0.102
Rg (nm)	2.194	2.190	2.183

**Table 8:** ADMET properties for the designed compounds and the reference compound.

Compounds	49	49n	49p	49x
**Pharmacokinetic and ADME properties**				
HIA (%)	97.575	97.494	97.534	97.873
BBB (Log BB)	−0.428	−0.249	−0.214	−0.218
P-glycoprotein substrate	Yes	No	No	No
CYP2C19 inhibitor	No	No	No	No
CYP2C9 inhibitor	No	No	No	No
CYP2D6 inhibitor	No	No	No	No
CYP3A4 inhibitor	No	No	No	No
**Toxicological properties**				
Mutagenic	No risk	No risk	No risk	No risk
Tumorigenic	No risk	No risk	No risk	No risk
Irritant	No risk	No risk	No risk	No risk
Reproductive effect	No risk	No risk	No risk	No risk

**Table 9:** Percentage of the identity of the three splits.

	Split 1 (%)					Split 2 (%)					Split 3 (%)				
Set	Total	AT	PT	Cal	Val	Total	AT	PT	Cal	Val	Total	AT	PT	Cal	Val
**Split 1 (%)**	100	100	100	100	100	25.5	25.6	35.9	0.0	25.0	32.7	36.8	35.0	50.0	0.0
**Split 2 (%)**						100	100	100	100	100	30.9	35.9	41.0	12.5	12.5
**Split 3 (%)**											100	100	100	100	100

**Table 10:** The detailed description of SMILES attributes.

SMILES Attributes	Description
S_k_	One symbol or two symbols that cannot be examined separately
SS_k_	Combination of two SMILES-atomes
SSS_k_	Combination of three SMILES-atomes
PAIR	Alliance of two descriptors NOSP ^a^ and BOND ^b^
HARD	Existence of some chemical element
Cmax	Number of rings
Omax	Number of oxygen atoms
Nmax	Number of nitrogen atoms

NOSP ^a^ descriptors are the structural features showing the presence or absence of nitrogen, oxygen, sulfur, phosphorus, while the BOND ^b^ descriptors respond to the presence or absence of double, triple, or stereochemical covalent bonds (=, #, @ or @@).

**Table 11:** List of key residues of SARS-CoV-2 Mpro.

Role in the SARS-CoV-2 Mpro	Residues
Substrate binding	HIS41, MET49, GLY143, SER144, HIS163, HIS164, MET165, GLU166, LEU167, ASN187, ARG188, GLN189, THR190, ALA191, GLN192
Dimerization	ARG4, SER10, GLY11, GLU14, ASN28, SER139, PHE140, SER147, GLU290, ARG298
Catalytic dyad	HIS41, CYS145

## Data Availability

Data is contained within the article and Supplementary Material.

## Supplementary Materials

The following supporting information can be downloaded at: https://www.mdpi.com/article/10.3390/ph16040608/s1. Figure S1: Superimposed view of native pose of co-crystallized ligand (black) with its X-ray pose (red) inside the CS of Mpro; Table S1: Split distribution, experimental pIC50 and calculated pIC50 for the three splits using TF2 (with W_IIC_ = 0.2); Table S2: Chemical structures of designed Mpro inhibitors; Table S3: Details of ligand–protein interactions of designed compounds within the CS of Mpro; Table S4: Details of ligand–protein interactions of designed compounds within the DIS of Mpro; Table S5: Chemical structure of the 55 dihydrophenanthrene derivatives and their corresponding IC50; Table S6: SMILES notation of the 55 dihydrophenanthrene derivatives and their pIC50.
