# Supplementary material for: Design of Potent Inhibitors Targeting the Main Protease of SARS-CoV-2 Using QSAR Modeling, Molecular Docking, and Molecular Dynamics Simulations
Source: Pharmaceuticals (Basel). 2023 Apr 18;16(4):608. doi: 10.3390/ph16040608 (PMC10146715; doi:10.3390/ph16040608)
Supplement: Supplementary file 1 [file pharmaceuticals-16-00608-s001.zip › pharmaceuticals-2308414-supplementary.pdf]

Supplementary Material to the manuscript entitled

Article

Design of Potent Inhibitors Targeting the Main Protease of SARS-CoV-2 Using QSAR Modeling, Molecular Docking, and Molecular Dynamics Simulations

Mehdi Oubahmane [1], Ismail Hdoufane [1], Christelle Delaite [2], Adlane Sayede [3], Driss Cherqaoui [1] and Achraf El Allali [4,*]

[1] Laboratory of Molecular Chemistry, Department of Chemistry, Faculty of Sciences Semlalia, BP 2390,
Marrakech 40000, Morocco; mehdi.oubahmane@ced.uca.ma (M.O.); ismail.hdoufane@edu.uca.ma (I.H.); cherqaoui@uca.ma (D.C.)

[2] Laboratoire de Photochimie et d'Ingénierie Macromoléculaires (LPIM), Ecole Nationale Supérieure de Chimie de Mulhouse, Université de Haute-Alsace, 68100 Mulhouse, France; christelle.delaite@uha.fr

[3] University Artois, CNRS, Centrale Lille, University Lille, UMR 8181, Unité de Catalyse et Chimie du Solide (UCCS), F-62300 Lens, France; adlane.sayede@univ-artois.fr

[4] African Genome Center, Mohammed VI Polytechnic University, Ben Guerir 43150, Morocco

* Correspondence: achraf.elallali@um6p.ma

Figure S1: Superimposed view of native pose of co-crystallized ligand (black) with its x-ray pose (red) inside the CS of Mpro.

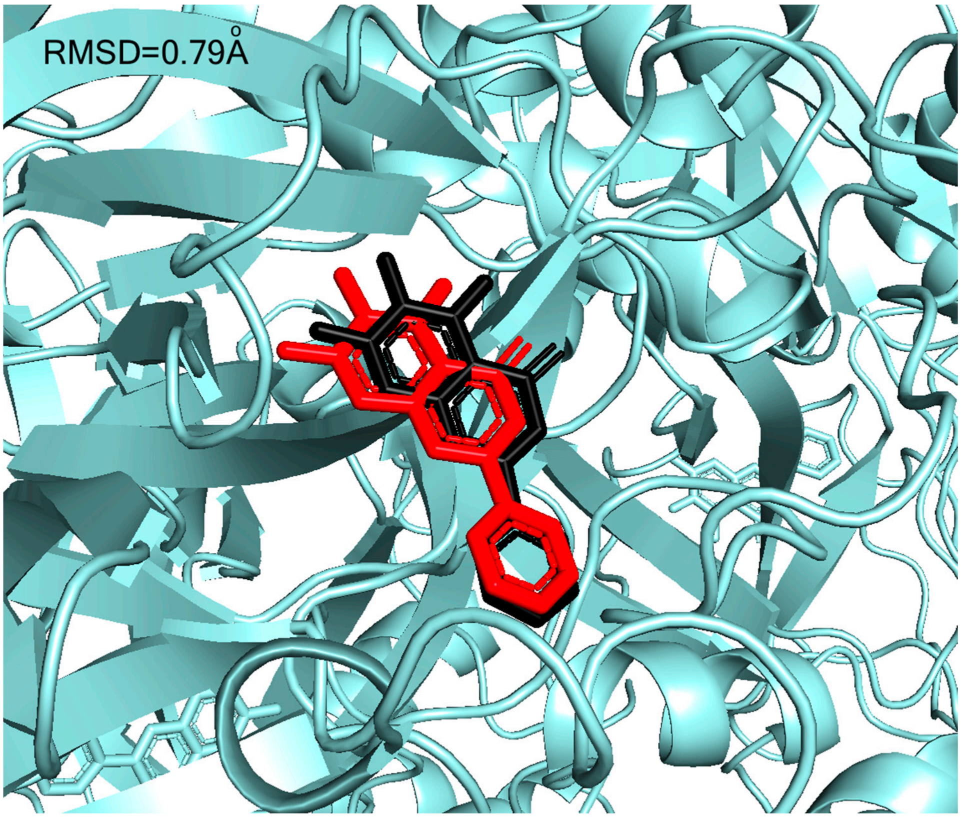

Table S1: Split distribution, experimental pIC_{50} and calculated pIC_{50} for the three splits using TF2 (with W_{IIC}=0.2).

ID	Exp.	Split[a]			Calculated pIC_{50}			Applicability domain		
	pIC_{50}	1	2	3	Split 1	Split 2	Split 3	Split 1	Split 2	Split 3
1	4.214	+	−	−	4.1428	4.1300	4.1929	Yes	Yes	Yes
2	4.481	+	−	*	4.2131	4.3113	4.2630	Yes	Yes	Yes
3	4.531	−	*	*	4.6411	4.3567	4.6561	Yes	Yes	Yes
4	5.043	−	#	−	4.5873	4.6349	4.8800	Yes	Yes	Yes
5	5.190	−	+	*	5.1855	5.1814	5.1846	Yes	Yes	Yes
6	4.714	+	+	+	4.9933	4.7377	5.0510	Yes	Yes	Yes
7	4.945	#	+	#	4.7985	4.9938	4.8746	Yes	Yes	Yes
8	4.000	−	−	−	4.1463	4.0711	4.2708	Yes	Yes	Yes
9	4.000	*	−	+	4.1666	4.0625	4.2863	Yes	Yes	Yes
10	4.000	−	#	+	4.0680	4.0934	4.2136	Yes	Yes	Yes
11	4.068	#	+	#	4.0680	4.0934	4.2136	Yes	Yes	Yes
12	4.000	+	+	+	4.1903	4.2836	4.3152	Yes	Yes	Yes
13	4.239	*	+	+	4.2022	4.0959	4.2421	Yes	Yes	Yes
14	4.269	+	*	−	4.2825	4.1926	4.3054	Yes	Yes	Yes
15	4.230	−	+	+	4.1458	4.1612	4.1659	Yes	Yes	Yes
16	4.166	−	*	−	4.2022	4.0959	4.2421	Yes	Yes	Yes
17	5.248	−	−	−	4.6983	4.7519	4.7947	Yes	Yes	Yes
18	4.175	+	+	*	4.1502	4.0096	4.2101	Yes	Yes	Yes
19	4.446	*	*	−	4.1502	4.0996	4.2101	Yes	Yes	Yes
20	4.733	+	−	+	4.2724	4.2897	4.3117	Yes	Yes	Yes
21	4.849	+	−	−	4.6208	4.7470	4.7209	Yes	Yes	Yes
22	4.922	−	−	+	4.7155	4.8018	4.7180	Yes	Yes	Yes
23	4.859	+	#	−	4.7288	4.7642	4.7430	Yes	Yes	Yes
24	4.000	+	−	−	4.0597	3.9386	4.0623	Yes	Yes	Yes
25	4.000	+	−	−	4.0477	3.9175	4.0262	Yes	Yes	Yes
26	4.000	−	#	#	3.9729	3.9334	4.0129	Yes	Yes	Yes
27	4.000	+	+	*	4.3913	4.0742	4.2889	Yes	Yes	Yes
28	4.000	+	−	+	4.3244	3.9341	4.1780	Yes	Yes	Yes
29	5.069	#	+	#	5.1427	5.2487	5.0898	Yes	Yes	Yes
30	5.609	−	#	−	5.7410	5.7953	5.7864	Yes	Yes	Yes
31	5.008	−	*	−	5.2449	5.1393	5.2338	Yes	Yes	Yes
32	5.320	−	+	+	5.1557	5.1861	5.1766	Yes	Yes	Yes
33	4.989	+	#	+	5.2160	5.2912	5.2186	Yes	Yes	Yes
34	5.479	+	−	+	5.2432	5.1299	5.2476	Yes	Yes	Yes
35	5.000	#	+	−	5.2746	5.1878	5.3179	Yes	Yes	Yes
36	4.950	+	#	−	5.0011	5.1196	5.0366	Yes	Yes	Yes
37	5.131	*	+	#	5.1074	5.0168	5.0161	Yes	Yes	Yes
38	5.280	+	−	*	5.2712	5.1206	5.2590	Yes	Yes	Yes
39	5.368	#	*	+	5.1820	5.1674	5.2018	Yes	Yes	Yes
40	5.565	−	−	+	5.1820	5.1674	5.2018	Yes	Yes	Yes
41	5.172	−	−	−	5.1926	5.1977	5.2477	Yes	Yes	Yes
42	5.556	+	+	*	5.2423	5.2725	5.2438	Yes	Yes	Yes
43	5.051	#	+	−	5.2695	5.2012	5.2728	Yes	Yes	Yes
44	5.177	*	*	−	5.2014	5.0097	5.3382	Yes	Yes	Yes
45	5.234	+	−	+	5.3909	5.2401	5.4139	Yes	Yes	Yes
46	5.437	#	*	#	5.3009	5.1691	5.3431	Yes	Yes	Yes

47	5.082	−	−	+	5.7673	5.7766	5.8117	Yes	Yes	Yes
48	5.480	#	−	−	5.6942	5.6731	5.7102	Yes	Yes	Yes
49	5.810	−	+	+	5.6913	5.7831	5.6733	Yes	Yes	Yes
50	5.742	−	−	+	5.1300	5.4103	5.1793	Yes	Yes	Yes
51	4.990	*	+	#	5.0950	4.8285	4.9463	Yes	Yes	Yes
52	5.268	−	+	+	5.1321	5.1449	5.0887	Yes	Yes	Yes
53	5.327	*	#	−	5.1321	5.1449	5.0887	Yes	Yes	Yes
54	4.971	−	+	*	5.1925	5.2499	5.1307	Yes	Yes	Yes
55	5.260	*	+	#	5.2511	5.1466	5.2300	Yes	Yes	Yes

[a] the signs represent the type of set to which each compound belongs in each split. +, −, #, and * represent AT, PT, Cal, and Val sets respectively.

Table S2: Chemical structures of designed Mpro inhibitors

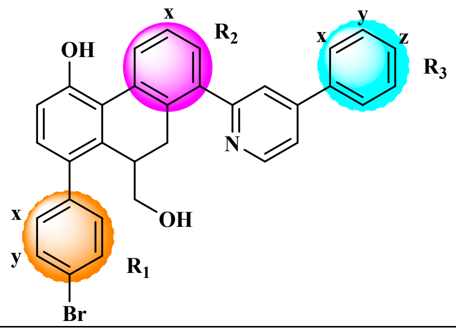

ID			Promoter of increase	Feature	pIC_{50} (CORAL)	pIC_{50} (GA-MLR)	Binding affinity in CS (Kcal/mol)
49					**5.78***	**5.65***	**−11.47**
49a	**R1**	x	C…C…(…	$-CH_3CH_3$	5.92	5.73	−11.77
49b			c…c…(…	$-CH_2CH_2$	5.95	5.74	−11.77
49c			c…c…c…	−Ph	6.44	6.18	**−10.62**
49d			C……… c…c…c…	−cyclopentdiene	6.14	5.80	−11.65
49e		y	C…C…(…	$-CH_3CH_3$	5.93	5.74	−11.96
49f			c…c…(…	$-CH_2CH_2$	5.86	5.70	−12.02
49g			c…c…c…	−Ph	6.44	5.99	−11.88
49h			C……… c…c…c…	−cyclopentdiene	6.29	5.67	−12.53
49i	**R2**	x	C…C…(…	$-CH_3CH_3$	5.91	5.79	−12.06
49j			c…c…(…	$-CH_2CH_2$	5.85	5.71	−12.21
49k			c…c…c…	−Ph	6.42	5.97	**−9.56**
49l			C……… c…c…c…	−cyclopentdiene	6.28	5.67	−11.92
49m	**R3**	x	C…C…(…	$-CH_3CH_3$	5.91	5.89	−12.46
49n			c…c…(…	$-CH_2CH_2$	**5.85**	**5.80**	**−12.63**
49o			c…c…c…	−Ph	6.42	6.21	−11.89
49p			C……… c…c…c…	−cyclopentdiene	**6.28**	**6.18**	**−12.98**
49q		y	C…C…(…	$-CH_3CH_3$	5.88	5.66	−12.18
49r			c…c…(…	$-CH_2CH_2$	5.82	5.69	−12.24
49s			c…c…c…	−Ph	6.39	6.01	−11.67
49t			C……… c…c…c…	−cyclopentdiene	6.22	5.70	−12.30
49u		z	C…C…(…	$-CH_3CH_3$	5.92	5.79	−12.44
49v			c…c…(…	$-CH_2CH_2$	5.89	**5.59**	−12.52
49w			c…c…c…	−Ph	6.44	5.99	−12.02
49x			C……… c…c…c…	−cyclopentdiene	**6.12**	**5.69**	**−12.85**

Table S3: Details of ligand−protein interactions of designed compounds within the CS of Mpro.

Compound	Binding affinity (Kcal/mol)	Category	Type	Residue	Distance (A⁰)
**49**	−11.47	Hydrogen Bond (HB)	Conventional HB	CYS44	2.40
				TYR54	2.36
				HIS164	1.90
			Carbon HB	THR26	3.62
			Pi−donor HB	CYS145	3.60
		Hydrophobic	Pi−sigma	ASN142	3.82
			Pi−Pi−stacked	HIS41	4.12
				HIS41	5.18
			Alkyl	LEU167	5.29
				PRO168	5.13
			Pi−alkyl	MET49	4.68
				MET49	5.15
				CYS44	5.30
				CYS145	4.22
		Other	Pi−sulfur	CYS44	5.29
				MET165	4.44
**49n**	−12.63	Hydrogen Bond (HB)	Conventional HB	CYS44	2.39
				TYR54	2.79
				HIS164	1.92
			Carbon HB	THR26	3.62
			Pi−donor HB	CYS145	3.86
				CYS145	3.99
		Hydrophobic	Pi−sigma	ASN142	3.78
			Pi−Pi−stacked	HIS41	4.21
			Alkyl	LEU167	5.09
				PRO168	5.08
				MET49	4.70
			Pi−alkyl	MET49	4.51
				MET49	5.17
		Other	Pi−sulfur	CYS44	5.45
				MET165	4.38
**49p**	−12.98	Hydrogen Bond (HB)	Conventional HB	HIS164	1.87
			Carbon HB	THR26	3.73
			Pi−donor HB	CYS145	3.89
				CYS145	4.06
		Hydrophobic	Pi−sigma	ASN142	3.80
			Pi−Pi−stacked	HIS41	4.42
				HIS41	5.06
			Alkyl	LEU167	4.98
				MET49	5.08
				PRO168	5.31
			Pi−alkyl	MET49	4.37
				MET49	5.08
		Other	Pi−sulfur	CYS44	5.38
				MET165	4.27
			Pi−lone pair	ARG188	2.94
**49x**	−12.85	Hydrogen Bond (HB)	Conventional HB	HIS164	1.90
				CYS44	2.41

		THR26	2.37
	Carbon HB	THR26	3.63
	Pi−donor HB	CYS145	3.63
Hydrophobic	Pi−sigma	ASN142	3.80
	Pi−Pi−stacked	HIS41	4.12
		HIS41	5.23
	Alkyl	LEU167	5.18
		PRO168	5.16
	Pi−alkyl	MET49	4.68
		MET49	5.16
		CYS44	5.32
		CYS145	4.25
Other	Pi−sulfur	CYS44	5.31
		MET165	4.41

Table S4: Details of ligand−protein interactions of designed compounds within the DIS of Mpro

Compound	Binding affinity (Kcal/mol)	Category	Type	Residue	Distance (A⁰)
**49**	−8.65	Hydrogen Bond (HB)	Conventional HB	VAL125	2.21
			Carbon HB	ARG4	2.84
		Hydrophobic	Pi−Pi T−shaped	PHE291	5.33
			Alkyl	ARG4	3.50
				MET6	4.79
			Pi−alkyl	LYS5	3.86
				LYS5	4.25
				LYS5	5.29
				ARG4	4.01
		Electrostatic	Pi−cation	LYS5	3.27
		Other	Pi−sulfur	MET6	5.79
**49n**	−9.18	Hydrogen Bond (HB)	Conventional HB	VAL125	2.33
				LYS5	2.29
			Carbon HB	ARG4	2.80
		Hydrophobic	Pi−Pi T−shaped	PHE291	5.51
			Alkyl	ARG4	3.51
				MET6	4.80
				LYS128	4.82
			Pi−alkyl	ARG4	4.01
				LYS5	3.86
				LYS5	4.30
				LYS5	5.43
				TYR126	4.77
		Electrostatic	Pi−cation	LYS5	3.29
		Other	Pi−sulfur	MET6	5.84
**49p**	−9.95	Hydrogen Bond (HB)	Conventional HB	VAL125	2.24
			Carbon HB	ARG4	2.82
		Hydrophobic	Alkyl	ARG4	3.50
				MET6	4.72
				CYS128	5.33
			Pi−alkyl	ARG4	4.02
				LYS5	3.82
				LYS5	4.47
				LYS5	5.37
				TYR126	4.67
		Electrostatic	Pi−cation	LYS5	3.46
		Other	Pi−sulfur	MET6	5.75
**49x**	−9.06	Hydrogen Bond (HB)	Conventional HB	LYS5	1.86
				LYS5	1.86
				LYS5	2.16
				GLN127	2.13
			Carbon HB	ARG4	2.74
				GLU290	3.20
		Hydrophobic	Pi−PI stacked	TYR126	3.73
			Alkyl	VAL125	4.50
				VAL125	4.70
				ALA7	4.08

		Pi–alkyl	ARG4	4.89
			LYS5	5.27
			LYS5	4.01
			LYS5	5.06
	Electrostatic	Pi–anion	GLU290	3.89
	Other	Pi–lone pair	LYS5	2.91

Table S5: Chemical structure of the 55 dihydrophenanthrene derivatives and their corresponding IC_{50}

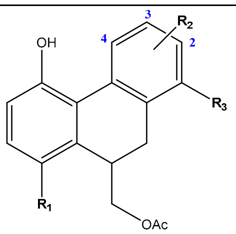

ID	R_1	R_2	R_3	IC_{50} (μM)
1	CH_3	H	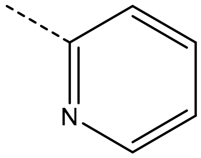	61.15
2	C_2H_5	H		33.06
3	Isopropyl	H		29.46
4	Cyclohexyl	H		9.06
5	4-Br phenyl	H		6.44
6	4-CN phenyl	H		19.32
7	Benzyl	H		11.39
8	CH_3	4-CH_3		>100
9	CH_3	4-OCH_3		>100
10	CH_3	4-F		>100
11	CH_3	4-Cl		85.58
12	CH_3	4-Br		>100
13	CH_3	3-CH_3		57.67
14	CH_3	2-CH_3		53.81
15	CH_3	H	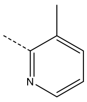	58.91
16	CH_3	H	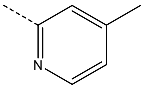	68.16
17	CH_3	H	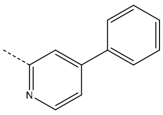	5.65
18	CH_3	H	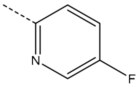	66.80
19	CH_3	H	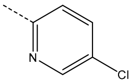	35.77
20	CH_3	H	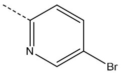	18.50
21	CH_3	H	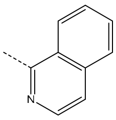	14.17
22	CH_3	H	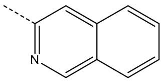	11.97
23	CH_3	H	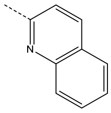	13.82
24	CH_3	H	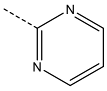	>100

25	CH_3	H	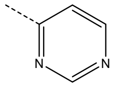	>100
26	CH_3	H	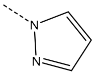	>100
27	CH_3	H	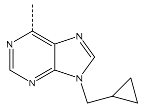	>100
28	CH_3	H	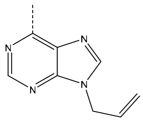	>100

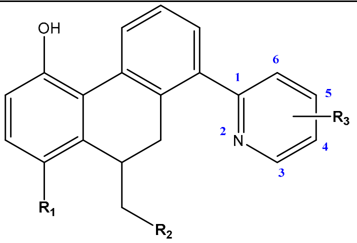

ID	R_1	R_2	R_3	IC_{50} (μM)
29	Cyclohexyl	OAc	5-Ph	8.54
30	4-Br Phenyl	OAc	5-Ph	2.46
31	4-Br Phenyl	OAc	5-CH_3	9.82
32	4-Br Phenyl	OAc	5-Cl	4.79
33	4-Br Phenyl	OAc	5-CN	10.26
34	4-Br Phenyl	OAc	5-CHO	3.32
35	4-Br Phenyl	OAc	5-NCH_3CH_3	10.00
36	4-Br Phenyl	OAc	5 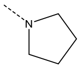	11.23
37	4-Br Phenyl	OAc	5 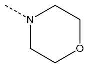	7.39
38	4-Br Phenyl	OAc	4-CH_3	5.25
39	4-Br Phenyl	OAc	4-F	4.29
40	4-Br Phenyl	OAc	4-Cl	2.72
41	4-Br Phenyl	OAc	4-Br	6.73
42	4-Br Phenyl	OAc	4-CN	2.78
43	4-Br Phenyl	OAc	4-CHO	8.89
44	4-Br Phenyl	OAc	4-methylsulfonyl	6.65
45	4-Br Phenyl	OAc	4-acetyl	5.83
46	4-Br Phenyl	OAc	4-NCH3CH3	3.66
47	4-Br Phenyl	OAc	4-Ph	8.28
48	4-Br Phenyl	OAc	4-N-Benzyl	3.31
49	4-Br Phenyl	OH	5-Ph	1.55
50	4-Br Phenyl	OH	5-CHO	1.81
51	4-Br Phenyl	OH	4-CH_3	10.23
52	4-Br Phenyl	OH	4-F	5.39
53	4-Br Phenyl	OH	4-Cl	4.71
54	4-Br Phenyl	OH	4-CN	10.68

55	4-Br Phenyl	OH	4-NCH_3CH_3	5.50

Table S6: SMILES notation of the 55 dihydrophenanthrene derivatives and their pIC_{50}

ID	SMILES	pIC_{50}
1	CC(=O)OCC1Cc2c(cccc2c2c(O)ccc(C)c21)c1ccccn1	4.214
2	CC(=O)OCC1Cc2c(cccc2c2c(O)ccc(CC)c21)c1ccccn1	4.481
3	CC(C)c1ccc(O)c2c3cccc(c3CC(COC(C)=O)c12)c1ccccn1	4.531
4	CC(=O)OCC1Cc2c(cccc2c2c(O)ccc(C3CCCCC3)c21)c1ccccn1	5.043
5	Brc1ccc(cc1)c1ccc(O)c2c3cccc(c3CC(COC(C)=O)c12)c1ccccn1	5.190
6	N#Cc1ccc(cc1)c1ccc(O)c2c3cccc(c3CC(COC(C)=O)c12)c1ccccn1	4.714
7	CC(=O)OCC1Cc2c(cccc2c2c(O)ccc(c3ccccc3)c21)c1ccccn1	4.945
8	CC(=O)OCC1Cc2c(ccc(C)c2c2c(O)ccc(C)c21)c1ccccn1	4.000
9	CC(=O)OCC1Cc2c(ccc(OC)c2c2c(O)ccc(C)c21)c1ccccn1	4.000
10	CC(=O)OCC1Cc2c(ccc(F)c2c2c(O)ccc(C)c21)c1ccccn1	4.000
11	CC(=O)OCC1Cc2c(ccc(Cl)c2c2c(O)ccc(C)c21)c1ccccn1	4.068
12	CC(=O)OCC1Cc2c(ccc(Br)c2c2c(O)ccc(C)c21)c1ccccn1	4.000
13	CC(=O)OCC1Cc2c(cc(C)cc2c2c(O)ccc(C)c21)c1ccccn1	4.239
14	CC(=O)OCC1Cc2c(c3ccccn3)c(C)ccc2c2c(O)ccc(C)c21	4.269
15	CC(=O)OCC1Cc2c(cccc2c2c(O)ccc(C)c21)c1nccccc1C	4.230
16	CC(=O)OCC1Cc2c(cccc2c2c(O)ccc(C)c21)c1cc(C)ccn1	4.166
17	CC(=O)OCC1Cc2c(cccc2c2c(O)ccc(C)c21)c1cc(ccn1)c1ccccc1	5.248
18	CC(=O)OCC1Cc2c(cccc2c2c(O)ccc(C)c21)c1ccc(F)cn1	4.175
19	CC(=O)OCC1Cc2c(cccc2c2c(O)ccc(C)c21)c1ccc(Cl)cn1	4.446
20	CC(=O)OCC1Cc2c(cccc2c2c(O)ccc(C)c21)c1ccc(Br)cn1	4.733
21	CC(=O)OCC1Cc2c(cccc2c2nccc3ccccc32)c2c(O)ccc(C)c21	4.849
22	CC(=O)OCC1Cc2c(cccc2c2cc3ccccc3cn2)c2c(O)ccc(C)c21	4.922
23	CC(=O)OCC1Cc2c(cccc2c2ccc3ccccc3n2)c2c(O)ccc(C)c21	4.859
24	CC(=O)OCC1Cc2c(cccc2c2c(O)ccc(C)c21)c1ncccn1	4.000
25	CC(=O)OCC1Cc2c(cccc2c2c(O)ccc(C)c21)c1ccncn1	4.000
26	CC(=O)OCC1Cc2c(cccc2c2c(O)ccc(C)c12)n1cccn1	4.000
27	CC(=O)OCC1Cc2c(cccc2c2ncnc3c2ncn3CC2CC2)c2c(O)ccc(C)c21	4.000
28	CC(=O)OCC1Cc2c(cccc2c2ncnc3c2ncn3CC=C)c2c(O)ccc(C)c21	4.000
29	CC(=O)OCC1Cc2c(cccc2c2c(O)ccc(C3CCCCC3)c21)c1cc(ccn1)c1ccccc1	5.069
30	Brc1ccc(cc1)c1ccc(O)c2c3cccc(c3CC(COC(C)=O)c12)c1cc(ccn1)c1ccccc1	5.609
31	Brc1ccc(cc1)c1ccc(O)c2c3cccc(c3CC(COC(C)=O)c12)c1cc(C)ccn1	5.008
32	Brc1ccc(cc1)c1ccc(O)c2c3cccc(c3CC(COC(C)=O)c12)c1cc(Cl)ccn1	5.320
33	Brc1ccc(cc1)c1ccc(O)c2c3cccc(c3CC(COC(C)=O)c12)c1cc(ccn1)C#N	4.989
34	Brc1ccc(cc1)c1ccc(O)c2c3cccc(c3CC(COC(C)=O)c12)c1cc(ccn1)C=O	5.479
35	Brc1ccc(cc1)c1ccc(O)c2c3cccc(c3CC(COC(C)=O)c12)c1cc(ccn1)N(C)C	5.000
36	Brc1ccc(cc1)c1ccc(O)c2c3cccc(c4nccc(c4)N4CCCC4)c3CC(COC(C)=O)c12	4.950
37	Brc1ccc(cc1)c1ccc(O)c2c3cccc(c4cc(ccn4)N4CCOCC4)c3CC(COC(C)=O)c12	5.131
38	Brc1ccc(cc1)c1ccc(O)c2c3cccc(c3CC(COC(C)=O)c12)c1ccc(C)cn1	5.280
39	Brc1ccc(cc1)c1ccc(O)c2c3cccc(c3CC(COC(C)=O)c12)c1ccc(F)cn1	5.368
40	Brc1ccc(cc1)c1ccc(O)c2c3cccc(c3CC(COC(C)=O)c12)c1ccc(Cl)cn1	5.565
41	Brc1ccc(cc1)c1ccc(O)c2c3cccc(c3CC(COC(C)=O)c12)c1ccc(Br)cn1	5.172
42	Brc1ccc(cc1)c1ccc(O)c2c3cccc(c3CC(COC(C)=O)c12)c1ccc(cn1)C#N	5.556
43	Brc1ccc(cc1)c1ccc(O)c2c3cccc(c3CC(COC(C)=O)c12)c1ccc(cn1)C=O	5.051
44	Brc1ccc(cc1)c1ccc(O)c2c3cccc(c3CC(COC(C)=O)c12)c1ccc(cn1)S(C)(=O)=O	5.177
45	Brc1ccc(cc1)c1ccc(O)c2c3cccc(c3CC(COC(C)=O)c12)c1ccc(cn1)C(C)=O	5.234
46	Brc1ccc(cc1)c1ccc(O)c2c3cccc(c3CC(COC(C)=O)c12)c1ccc(cn1)N(C)C	5.437
47	Brc1ccc(cc1)c1ccc(O)c2c3cccc(c3CC(COC(C)=O)c12)c1ccc(cn1)c1ccccc1	5.082
48	Brc1ccc(cc1)c1ccc(O)c2c3cccc(c3CC(COC(C)=O)c12)c1ccc(cn1)NCc1ccccc1	5.480

49	Brc1ccc(cc1)c1ccc(O)c2c3cccc(c3CC(CO)c12)c1cc(ccn1)c1ccccc1	5.810
50	Brc1ccc(cc1)c1ccc(O)c2c3cccc(c3CC(CO)c12)c1cc(ccn1)C=O	5.742
51	Brc1ccc(cc1)c1ccc(O)c2c1c(CO)cc1c(cccc21)c1ccc(C)cn1	4.990
52	Brc1ccc(cc1)c1ccc(O)c2c3cccc(c3CC(CO)c12)c1ccc(F)cn1	5.268
53	Brc1ccc(cc1)c1ccc(O)c2c3cccc(c3CC(CO)c12)c1ccc(Cl)cn1	5.327
54	Brc1ccc(cc1)c1ccc(O)c2c3cccc(c3CC(CO)c12)c1ccc(cn1)C#N	4.971
55	Brc1ccc(cc1)c1ccc(O)c2c3cccc(c3CC(CO)c12)c1ccc(cn1)N(C)C	5.260
